# Zygotic *Porcn* Paternal Allele Deletion in Mice to Model Human Focal Dermal Hypoplasia

**DOI:** 10.1371/journal.pone.0079139

**Published:** 2013-11-01

**Authors:** Steffen Biechele, Hibret A. Adissu, Brian J. Cox, Janet Rossant

**Affiliations:** 1 Program in Developmental and Stem Cell Biology, Hospital for Sick Children Research Institute, Toronto, Ontario, Canada; 2 Department of Molecular Genetics, University of Toronto, Toronto, Ontario, Canada; 3 Physiology & Experimental Medicine, the Hospital for Sick Children, Toronto, Ontario, Canada; 4 Department of Laboratory Medicine and Pathobiology, Faculty of Medicine, University of Toronto, Toronto, Ontario, Canada; IGBMC/ICS, France

## Abstract

In mouse and humans, the X-chromosomal *Porcupine homolog* (*Porcn*) gene is required for the acylation and secretion of all 19 Wnt ligands, thus representing a bottleneck in the secretion of Wnt ligands. In humans, mutations in *PORCN* cause the X-linked dominant syndrome Focal Dermal Hypoplasia (FDH, OMIM#305600). This disorder is characterized by ecto-mesodermal dysplasias and shows a highly variable phenotype, potentially due to individual X chromosome inactivation patterns. To improve the understanding of human FDH, we have established a mouse model by generation of Porcn heterozygous animals carrying a zygotic deletion of the paternal allele. We show that heterozygous female fetuses display variable defects that do not significantly affect survival in the uterus, but lead to perinatal lethality in more than 95% of females. Rare survivors develop to adulthood and display variable skeletal and skin defects, representing an adult zygotic mouse model for human FDH. Although not frequently reported in humans, we also observed bronchopneumonia, rhinitis, and otitis media in these animals, suggesting a potential link between *Porcn* function and the normal development of ciliated cells in these tissues.

## Introduction

Wnt ligands are secreted proteins that bind to Frizzled receptors, activating several interconnected downstream signaling pathways that are required both for embryonic development as well as adult tissue homeostasis [1]. The X-chromosomal *Porcupine homolog* (*Porcn*) gene encodes a membrane-bound O-acyl transferase (MBOAT) that is required for the acylation of all 19 Wnt ligands encoded in the mammalian genome [2,3]. Acylation of Wnts has been shown to be required for secretion from the producing cells as well as for binding of Frizzled receptors on signal-receiving cells [4-6]. Non-acylated Wnt ligands hence cannot activate downstream signaling targets. Numerous mutations in WNT pathway components, including 6 *WNT* ligand genes, have been associated with human diseases and developmental defects [7]. 

Similar to mice, humans carry a single *PORCN* gene on the X chromosome (Xp11.23). Mutations in human *PORCN* cause FDH (Goltz Syndrome, OMIM#305600) [8,9], an X-linked dominant disorder characterized by dysplasias in ecto-mesodermal tissues. Phenotypically, FDH is characterized by patchy, hypoplastic skin, often along the lines of Blaschko [10,11]. Other features of the syndrome include digital abnormalities, microphthalmia, hypodontia, kidney abnormalities, abdominal wall defects, skeletal abnormalities, and reduced bone density [12]. The majority of FDH patients are heterozygous females, which are mosaic for PORCN function due to X chromosome inactivation (XCI). Male FDH patients represent approximately 10% of observed cases and carry post-zygotic mutations, creating a functional mosaicism similar to heterozygous females. The only exception published to date is a male Klinefelter/FDH patient (47,XXY) without detectable mosaicism for the *PORCN* mutation [13]. Similar to females carrying zygotic mutations, the second X chromosome carrying a wildtype *PORCN* allele leads to functional mosaicism due to XCI. Both the absence of zygotic mutant males or homozygous female patients, along with mouse data showing a requirement for *Porcn* in gastrulation [14-16], have led to the conclusion that human hemizygous *PORCN* mutants are embryonic lethal. In contrast, heterozygous females exhibit a variable phenotype, most likely due to differences in individual XCI patterns and frequencies. 

To date, three studies have used Porcn ablation in mice to model human FDH. Barrott et al. used epiblast-specific deletion of the maternal allele to generate heterozygous female animals [15]. Consistent with the human phenotype, these fetuses exhibited variable digital abnormalities, large areas of dermal atrophy and body wall closure defects. Using tissue-specific Porcn ablation, this study further showed that *Porcn* is required in both mesenchyme and ectoderm of the developing limb, but suggests a major role for *Porcn* in the ectoderm [15]. Postnatal phenotypes could only be assessed superficially due to perinatal lethality of the animals. Similarly, Liu et al. observed limb shortening and syndactyly when *Porcn* was deleted in the surface ectoderm of the developing limb [17]. Skin-specific deletion further recapitulated the human dermal hypoplasia with associated alopecia. Taking advantage of chimera formation as a proxy for XCI, this study furthermore observed defects in reproductive tracts and kidneys [17]. Addressing early embryonic phenotypes in detail and consistent with the above studies [15,17], we have previously shown that zygotic Porcn ablation in males causes embryonic lethality at gastrulation stages, whereas heterozygous females carrying a maternal allele deletion die at midgestation stages due to an extra-embryonic requirement for *Porcn* [16]. As *Porcn* and *Wls* (*Gpr177*) both play essential roles in Wnt secretion [18], tissues-specific ablations of *Wls* have recapitulated the tissue-specific *Porcn* phenotypes [19-21].

While such tissue-specific ablations provide valuable insights into FDH phenotypes, they do not recapitulate the XCI-mediated mosaicism of heterozygous females. In contrast, chimera formation approaches recapitulate mosaicism, but are likely biased by the developmental potential of the embryonic stem cell (ESC) lines used and complicated by sex chimerism (i.e. mutant male ESCs in female host embryos). Thus, in order to generate a mouse model that more accurately reproduces the X-linked genetics and inheritance of the human disease, we generated mice carrying a zygotic deletion of the paternal *Porcn* allele. We observed similar phenotypes at fetal stages as previous studies whilst also encountering significant perinatal lethality [15]. We were also, however, able to assess the phenotype of rare adult heterozygous females. In addition to dermal and skeletal defects, we observed rhinitis, otitis and bronchopneumonia in these mice. This combination of lesions is suggestive of a defect in ciliated cell development or cilia function, and may represent a novel aspect of human FDH.

## Results

### Embryonic defects cause perinatal lethality

**Figure 1:**
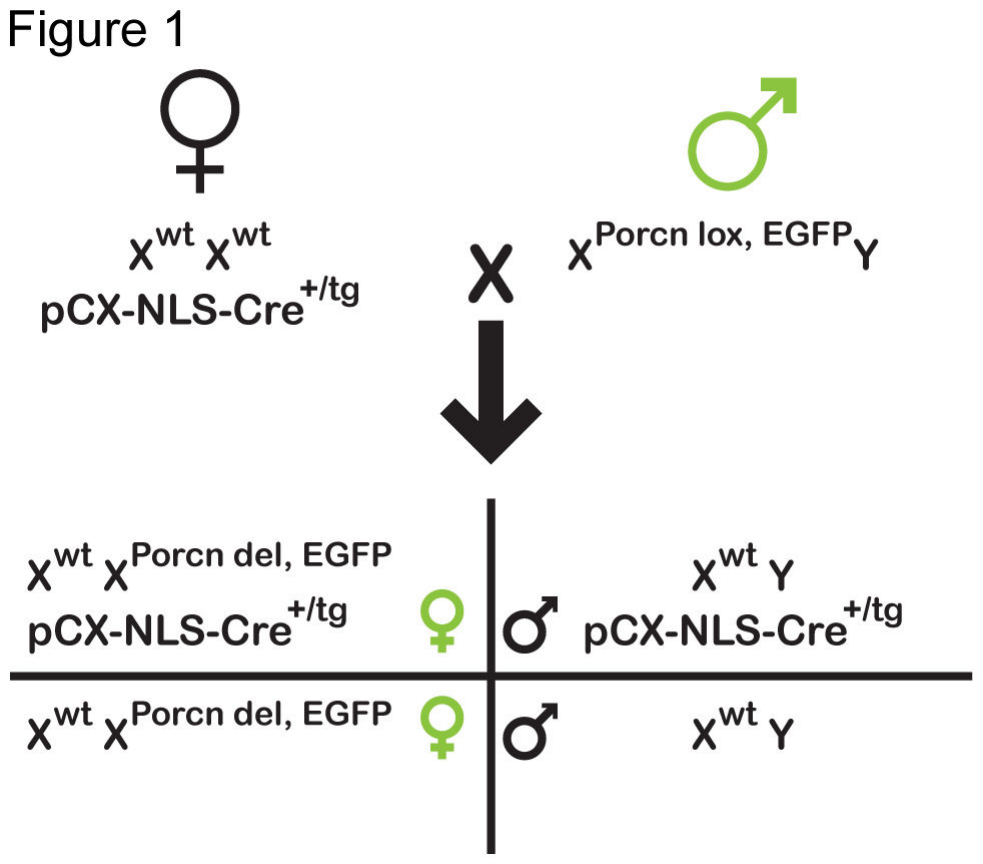
Schematic outline of the genetic strategy to generate *Porcn* ^*+/del*^ females. Maternally transmitted, ubiquitously expressed Cre recombinase (pCX-NLS-Cre transgene) is loaded into eggs, leading to the zygotic deletion of a paternally transmitted Porcn floxed allele upon fertilization independent of transgene transmission. A X-linked green fluorescent protein (GFP) transgene in cis to the floxed Porcn allele was used to identify female fetuses and pups. Due to XCI, functionally mutant cells express GFP.

We have previously shown that zygotic deletion of the maternal *Porcn* allele in female mouse embryos causes failure in chorio-allantoic fusion and embryonic lethality by embryonic day (E) 11.5 [16]. In order to establish a postnatal model for human FDH, we generated heterozygous females by zygotic deletion of the paternal allele (*Porcn* ^*+/del*^), using the ubiquitously expressed pCX-NLS-Cre transgene transmitted through the female germline (Figure 1), which were maintained on an outbred (ICR) background. An X-linked EGFP transgene in cis to the mutant allele was used to identify mutant cells in female fetuses and female neonates [22]. In mice and humans, XCI is random in the embryo proper, but imprinted in extra-embryonic tissues, leading to monoallelic expression from the maternal X chromosome [23,24]. Paternal allele mutant females thus have functionally wild-type extra-embryonic tissues.

**Figure 2:**
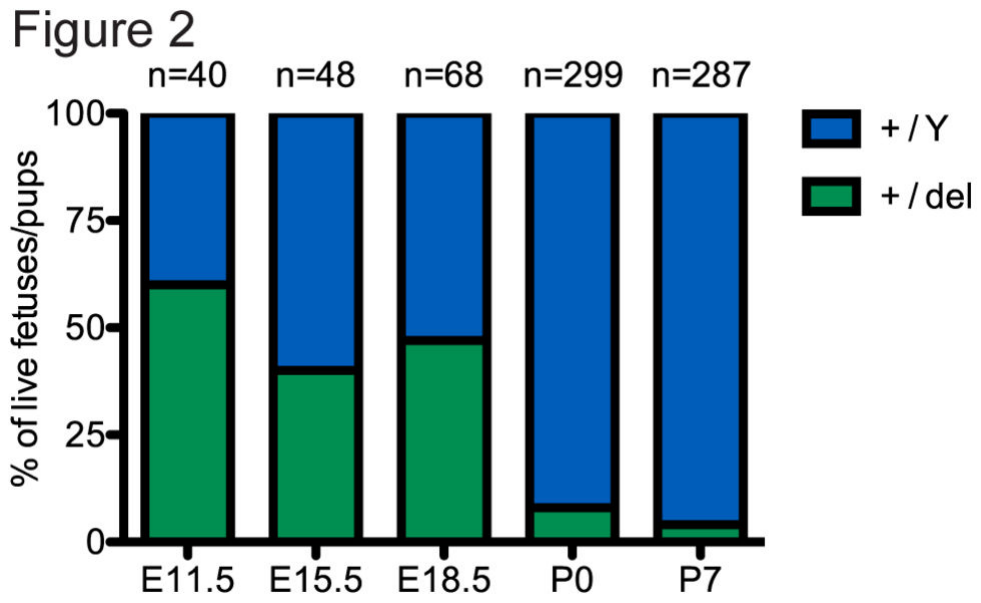
Analysis of survival of *Porcn* ^*+/del*^ fetuses and neonates. At fetal stages (E11.5 to E18.5) *Porcn* ^*+/del*^ females were observed at the expected Mendelian frequency (50%). At birth (P0) only 7.4% of the live neonates observed were female. Lethality in the first week of life further reduced this frequency to 3.5% (10 out of 277 expected) at postnatal day 7 (P7).

Despite the functional rescue of placental phenotypes, we noted that heterozygous females were underrepresented in newborn litters and a large percentage of these mice were lost due to perinatal mortality (Figure 2). However, by breeding larger numbers of mice, rare survivors were identified. Despite observations of occasional fetal lethality, we were able to observe *Porcn* ^*+/del*^ female fetuses up to E18.5 with no significant deviations from the expected frequency (50%, Figure 2), suggesting that the majority of malformations observed does not impinge on embryonic survival. Assessment of litters at birth (P0), however, revealed that most *Porcn* ^*+/del*^ females die perinatally and are cannibalized by the mothers, as only 7.4% of live pups were female (n=22/299,Figure 2). By postnatal day 7 (P7), only 3.5% female pups were alive (n=10/287), indicating a high rate of perinatal lethality (Figure 2). No unusual lethality was observed past P7. 

**Figure 3:**
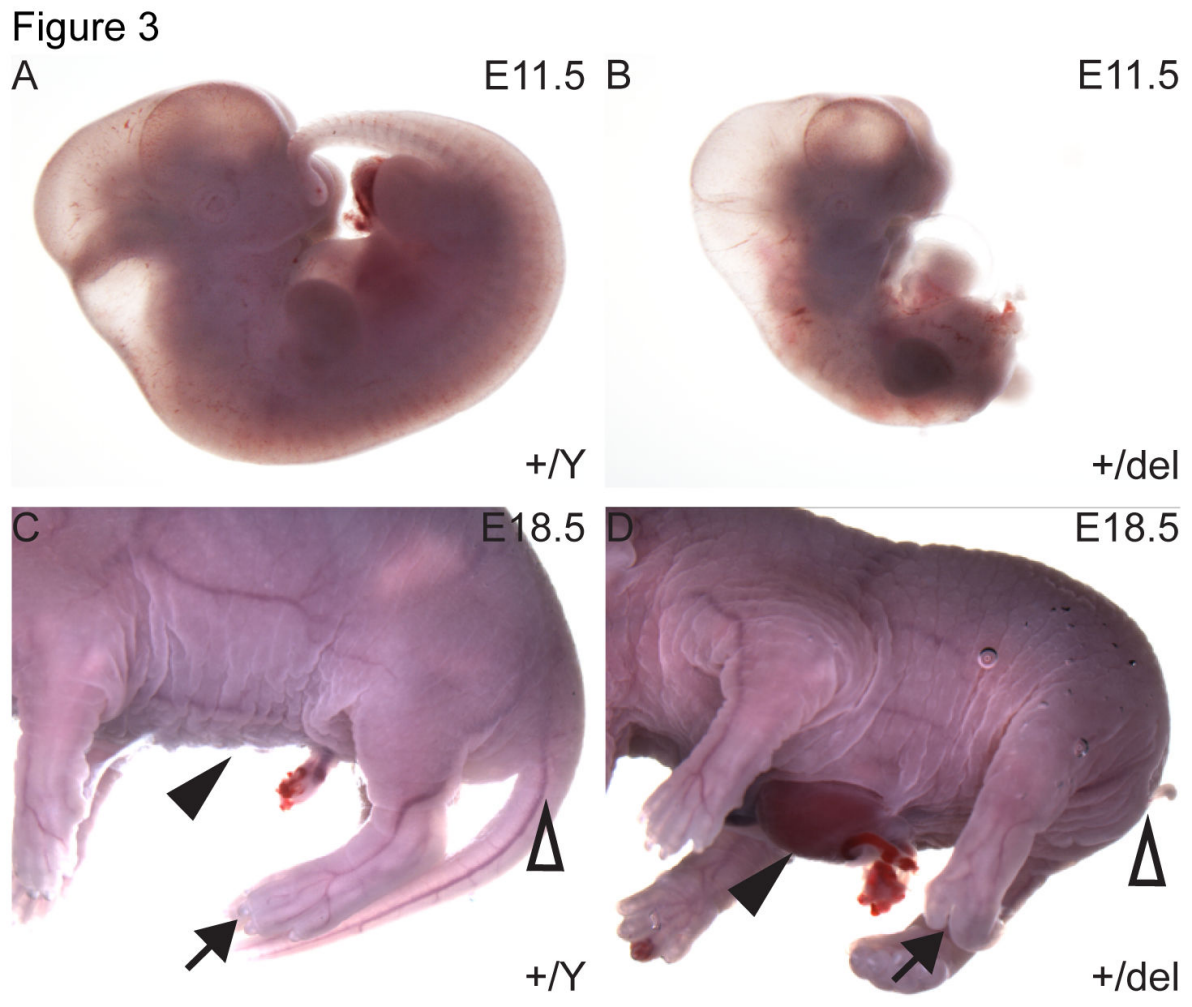
Gross morphological abnormalities in *Porcn* ^*+/del*^ fetuses. At E11.5, heterozygous females with posterior truncations could be observed (B), while wildtype *Porcn* ^*+/Y*^ littermates (A) developed normally. Just prior to birth (E18.5), several *Porcn* ^*+/del*^ females (D) displayed defects in body wall closure (arrowhead), digital abnormalities (arrow) and lack of tail (open arrowhead). Male littermates never displayed these defects (C).

**Figure 4:**
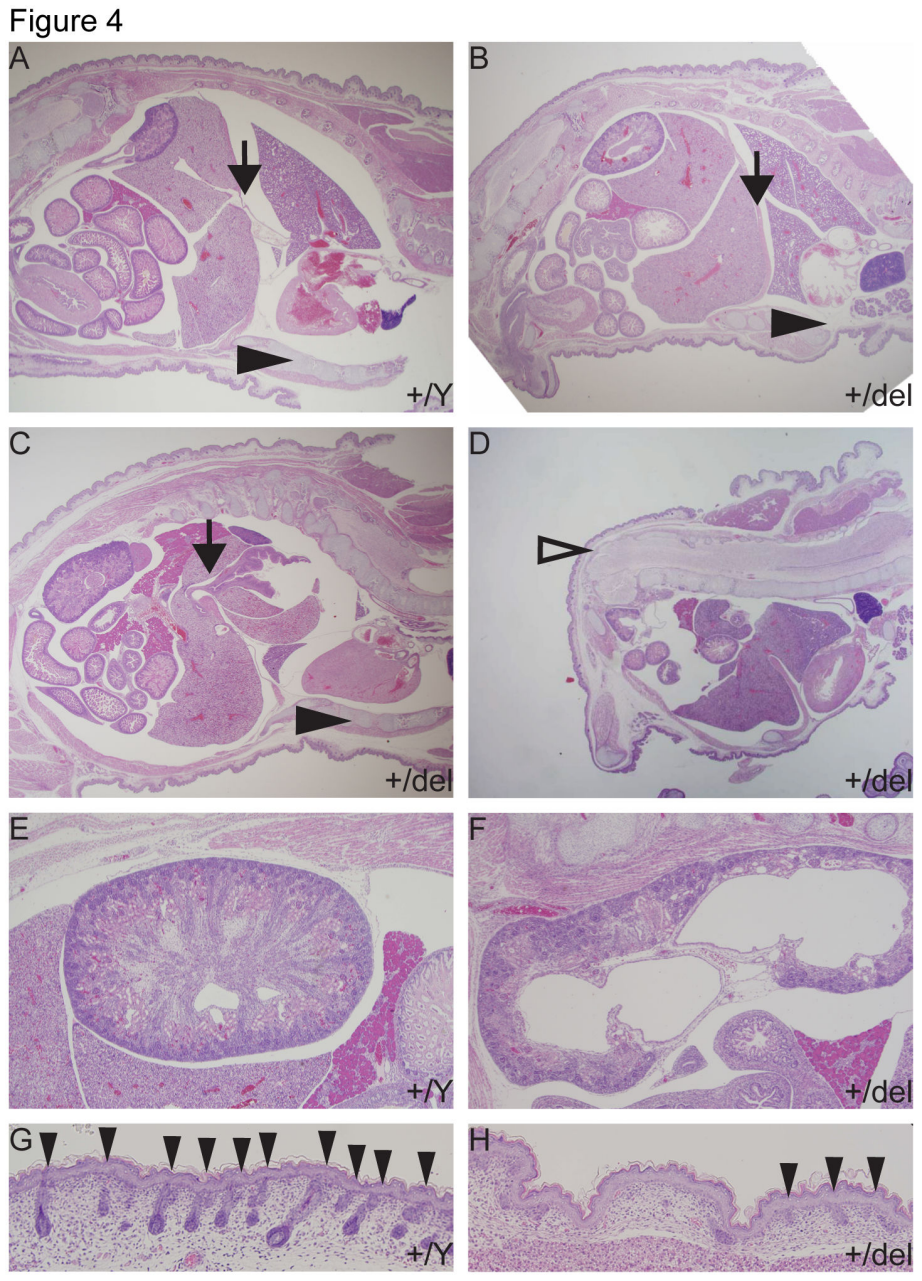
Histological analysis of *Porcn* ^*+/del*^ females at E18.5. At E18.5, in contrast to control *Porcn* ^*+/Y*^ littermates (A), several *Porcn* ^*+/del*^ females exhibited body wall closure defects (B, n=5/9), diaphragmatic hernias (C, n=5/9), and signs of spina bifida (D, open arrowhead, n=1/9). Arrows indicate the diaphragm. Arrowheads indicate the anterior body wall. Approximately 45% (n=4/9) heterozygous females displayed signs of severe kidney disease, such as hydronephrosis (F), which was not observed in control littermates (E). The skin of the majority of *Porcn* ^*+/del*^ fetuses (H, n=6/9) displayed signs of focal dermal hypoplasia as evidenced by reduction/absence of adnexa (arrowheads) (G,H).

As noted in human FDH as well as previous observations in mice [15], we found a wide spectrum of defects during fetal development of *Porcn* ^*+/del*^ females. Typical defects included posterior truncations (Figure 3A, B) reminiscent of *Cdx1::Cre* deletions of the downstream Wnt signaling component *Ctnnb1* [25], body wall closure defects and defects in tail development (Figure 3C, D), as well as craniofacial defects (data not shown). We additionally observed variable limb development defects including syndactyly, polydactyly, oligodactyly with features of ectrodactyly, and more severe malformations such as absence of feet and digits (*peromelia*). In order to investigate the cause of perinatal lethality in more detail, we assessed midsagittal and parasagittal sections of fetuses just prior to birth (E18.5). While some *Porcn* ^*del/+*^ fetuses (n=2/9) and *Porcn* ^*+/Y*^ littermates (n=2/2) showed no obvious abnormalities (Figure 4A), the majority of fetuses (n=7/9) exhibited multiple defects potentially responsible for the observed perinatal lethality. These individually variable defects could be grouped into four categories; thoracic body wall defects (n=5/9, Figure 4B), diaphragmatic hernias with abdominal organs protruding into the thoracic cavity (n=5/9, Figure 4C), kidney defects such as hydronephrosis and hydroureter (n=4/9, Figure 4E, F), and midline closure defects (Figure 3 D). We furthermore observed a high frequency of fetuses with focal dermal hypoplasia (n=6/9, Figure 4G, H), which can be excluded as cause of lethality, but is the name-giving feature of the human disease FDH. Thoracic body wall defects and diaphragmatic hernias are likely to impair lung function and compromise postnatal survival. Moreover, severe kidney defects can also cause lethality within the first days of life. Together with midline closure defects that expose organs to the exterior, these defects explain the perinatal lethality observed in *Porcn* ^*+/del*^ females.

### Rare adult Porcn^+/del^ females as a model for human FDH

**Figure 5:**
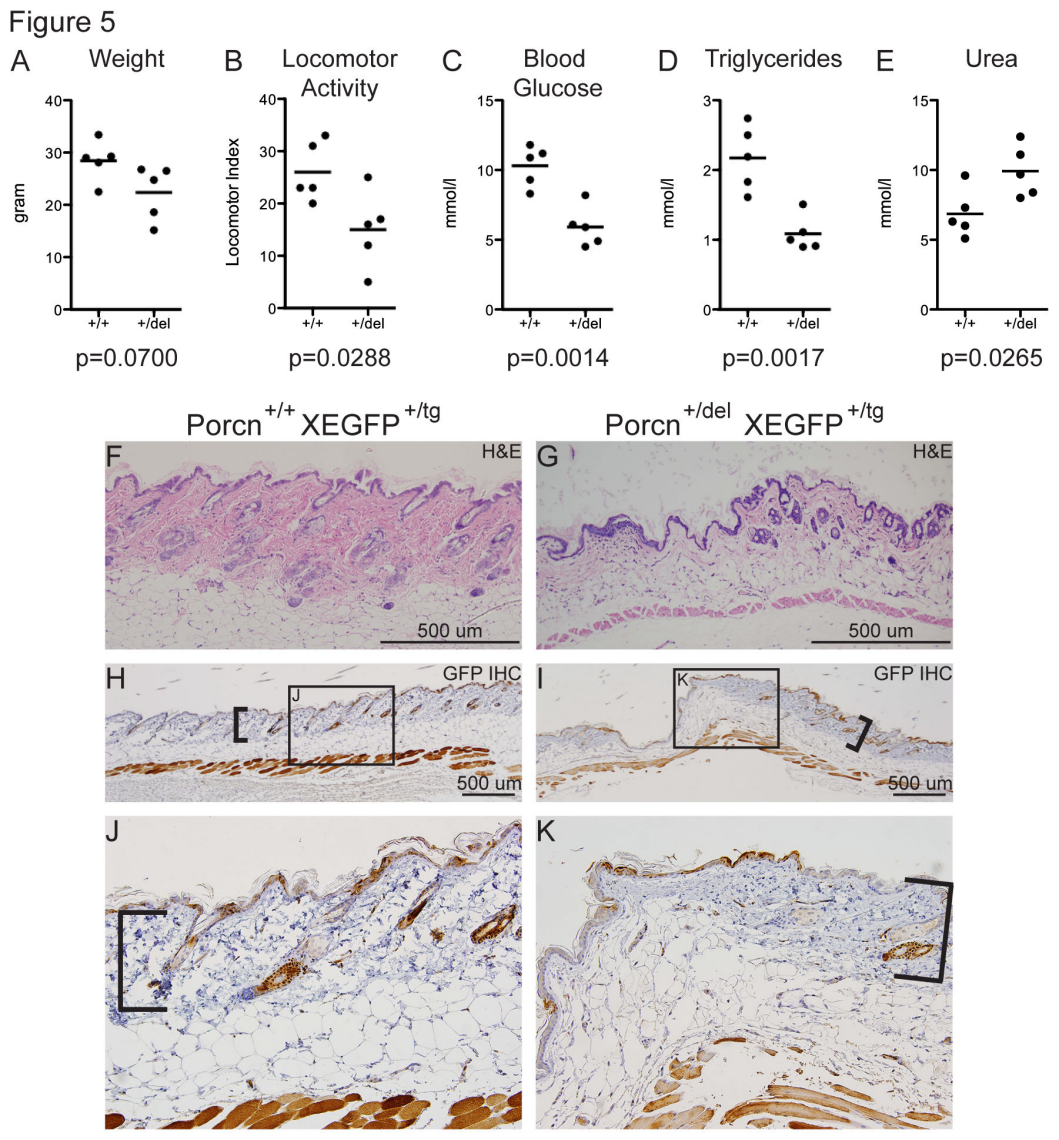
Adult *Porcn* ^*+/del*^ females as a model for FDH. Adult *Porcn* ^*+/del*^ exhibit reduced weight and locomotor activity compared to control *Porcn* ^*+/+*^ females (A, B). Blood glucose and triglyceride levels were reduced (C, D), whereas urea levels in the blood were increased (E). Heterozygous females further displayed FDH characteristic focal dermal hypoplasia with reduction/absence of dermal collagen and adnexal hypoplasia/aplasia (H&E staining, F, G). Functionally mutant cells are labeled by an X-linked GFP transgene in cis to the deleted allele. Immunohistochemistry for GFP (GFP IHC) did not reveal major differences in GFP expression patterns in the skin of heterozygous or control animals. Figures 5 A-E were analyzed by unpaired student’s t-test.

To establish the relationship between our FDH model mice and human FDH, we phenotyped five adult *Porcn* ^*+/del*^ *; XEGFP* ^*+/tg*^ *; pCX-NLS-Cre* ^*tg/+*^ females at nine to ten weeks of age. As the genetic strategy did not generate female control littermates, we used *Porcn* ^*+/+*^ *; XEGFP* ^*+/tg*^ *; pCX-NLS-Cre* ^*tg/+*^ females on the same genetic background as controls. Compared to control females, *Porcn* ^*+/del*^ females had a reduced bodyweight (p=0.07) and significantly reduced locomotor activity (Figure 5A, B), indicating poor clinical condition. Blood glucose and triglyceride levels were also significantly reduced (Figure 5C, D), potentially explaining the lethargy observed during locomotor activity testing. We furthermore observed increased blood urea levels (Figure 5E), pointing towards kidney defects. Despite this observation, kidney morphology and urinalysis were normal, suggesting a pre-renal cause such as reduced glomerular filtration due to dehydration.

Similar to observations made at E18.5, some females (2/5) had skin lesions. Consistent with a previous report [15] and the human phenotype, these lesions were characterized by a reduction in dermal collagen with segmental absence of hair follicles and associated structures (adnexal aplasia, Figure 5F, G). In order to characterize which cells within the lesions had an active mutant X chromosome, skin sections from affected regions were stained for EGFP, which labels functionally mutant cells. EGFP expression was equally mosaic in both normal and affected regions of the skin (Figure 5H-K), suggesting that the requirement for Wnt signaling is not confined to a readily identifiable cell source and is not cell-autonomous. 

### Skeletal defects in Porcn^+/del^ females

**Figure 6:**
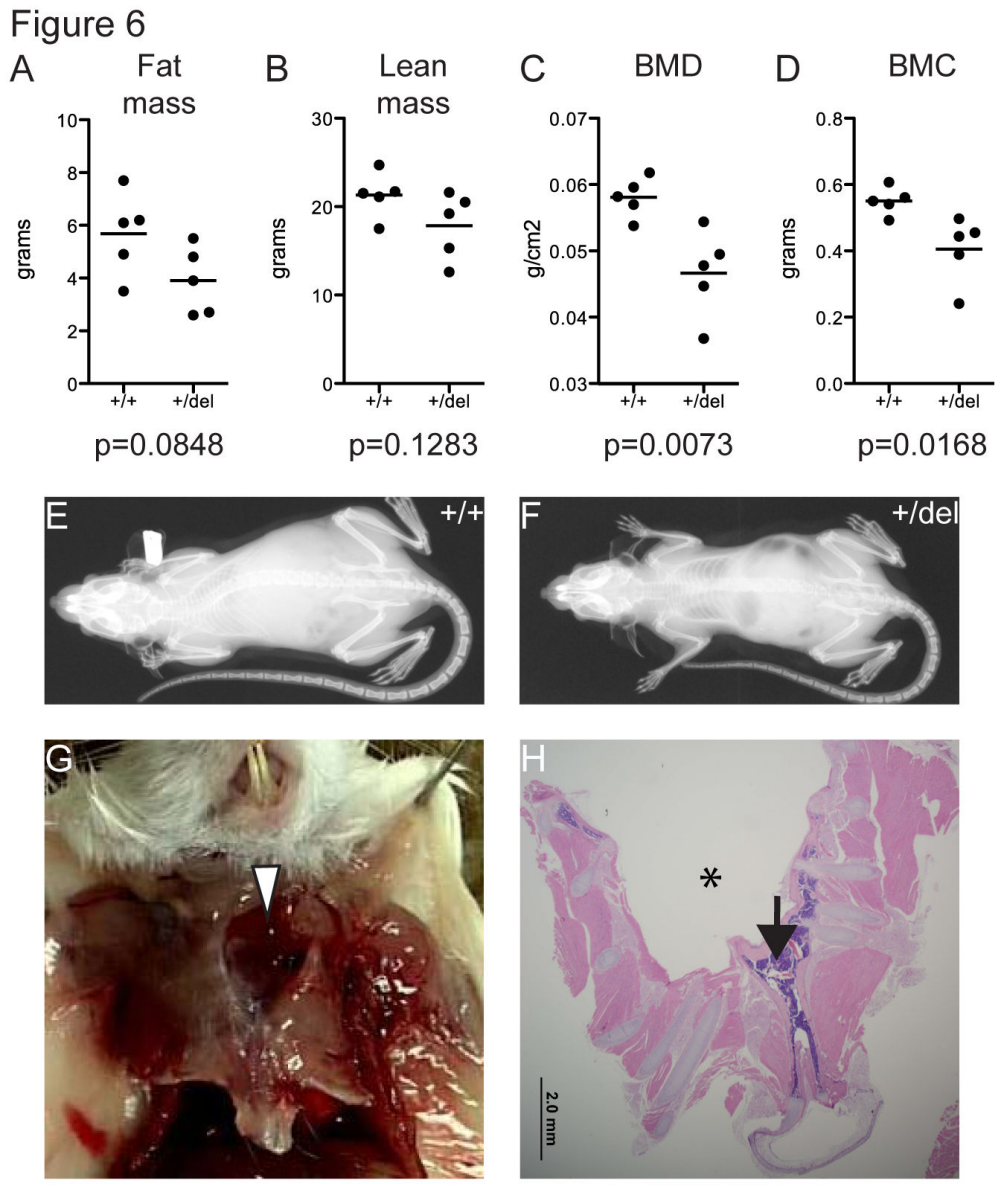
Body composition and skeletal phenotypes in adult *Porcn* ^*+/del*^ females. Adult *Porcn* ^*+/del*^ and *Porcn* ^*+/+*^ females were subjected to X-ray imaging and body composition analysis. While fat mass (A) and lean mass (B) was not significantly different (t-test), bone mineral density (BMD, C) and bone mineral content (BMC, D) were significantly reduced in heterozygous females. X-ray imaging was unremarkable in both control (E) and heterozygous females (F). One out of the five analyzed heterozygous female exhibited a sternal gap (G, white arrowhead). Consistent with duplication of the sternal skeleton, osseous structures on either side were enveloped by periosteal tissue (H). Arrow indicates the border between left and right ribcage and the sternal gap is indicated by asterisk.

As human patients frequently present with skeletal abnormalities and reduced bone density [26,27], we performed body composition analyses, X-ray imaging and necropsies on *Porcn* ^*+/del*^ females. While we could not detect significant changes in fat and lean mass (Figure 6A, B), the bone mineral density (BMD) and bone mineral content (BMC) of mutant mice were significantly reduced (Figure 6C, D). X-ray imaging was largely unremarkable (Figure 6E, F), although necropsies identified one mouse with a thoracic body wall defect; the thorax exhibited a 10 mm wedge-shaped gap in the sternal bone (Figure 6G). The sternal osseous and cartilaginous structures on either side of the defect were each enveloped by differentiated periosteal and perichondrial tissue consistent with duplication of the sternal skeleton (Figure 6H). Strikingly, human FDH patients with split sternum have also been observed [27,28], highlighting the similarities between the mouse and human phenotypes.

### Novel observations in the FDH mouse model

**Figure 7:**
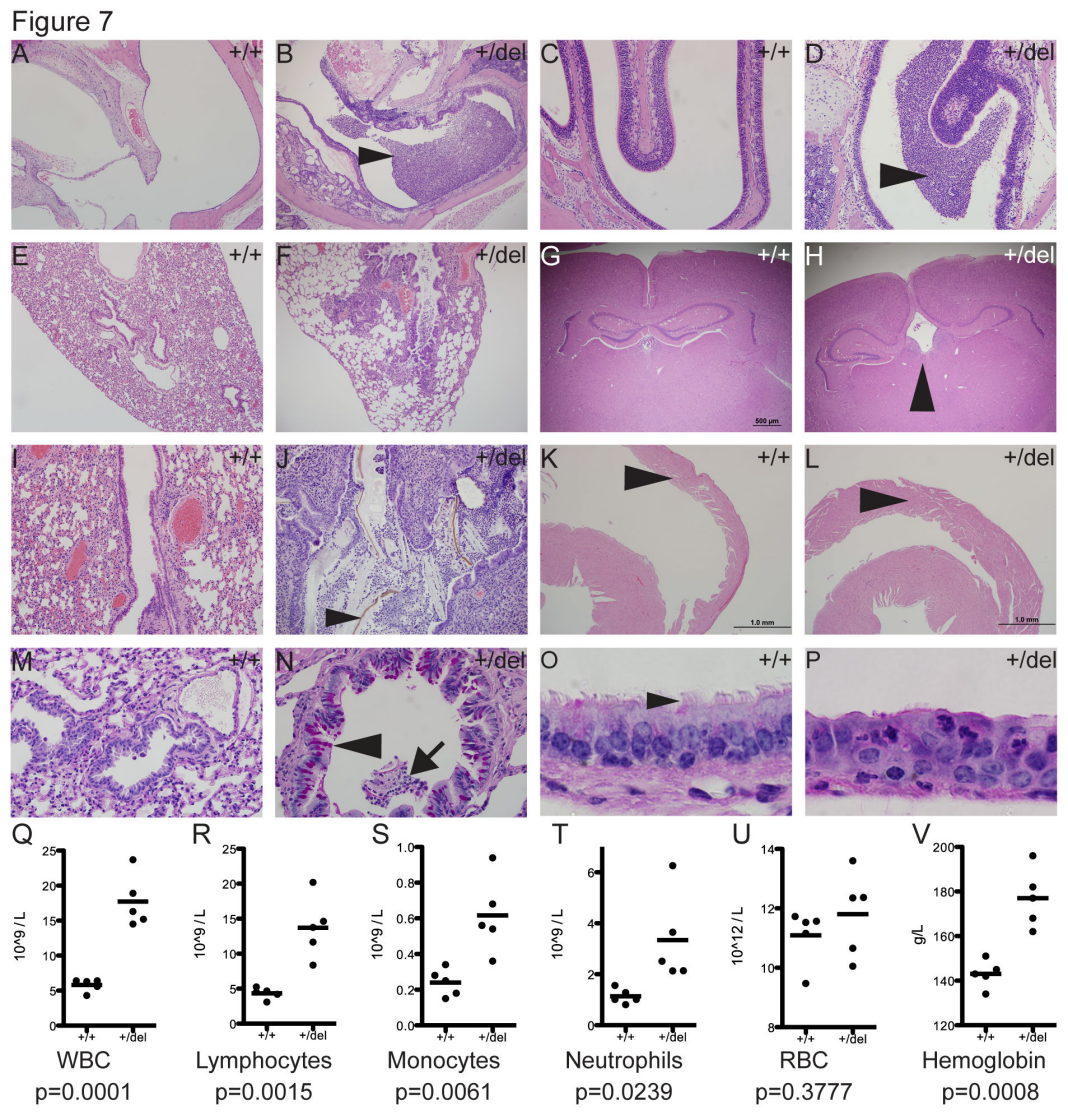
Novel observations in *Porcn* ^*+/del*^ FDH mouse model. In contrast to control *Porcn* ^*+/+*^ animals, adult *Porcn* ^*+/del*^ females exhibit lesions suggestive of ciliary defects (A-H). Mutants exhibited otitis media (n=4/5, B), rhinitis (n=2/5, D), and bronchopneumonia (n=3/5, F). Mild hydrocephalus was observed in 2/5 *Porcn* ^*+/del*^ females (H). Arrowhead indicates enlarged third ventricle (H). Bronchopneumonia was characterized by large numbers of inflammatory cells and plant material/bedding in the bronchioles (arrowhead, J), and was accompanied by mild right ventricular hypertrophy (arrowhead, L). Mutant bronchiole epithelia had increased numbers of goblet cells (N, pink cells, arrowhead) in areas of inflammation as indicated by the presence of intraluminal inflammatory cells (N, arrow). Tracheal epithelia of mutants were mildly disorganized (P) and lacked cilia in segments of up to 200 μm long compared to controls (O, arrowhead). Consistent with chronic active inflammation, hematology profiles showed significant increases in white blood cells (WBC, Q), lymphocytes (R), monocytes (S), and neutrophils (T). Red blood cell (RBC) counts (U) and hemoglobin concentration (V) were also elevated. Blood cell counts were analyzed by unpaired student’s t-test. Figures A-L: Hematoxylin & Eosin (H&E) stained sections. Figures M-P: Periodic Acid-Schiff (PAS) stained sections.

In contrast to control animals, adult *Porcn* ^*+/del*^ females exhibited combinations of mucociliary clearance defects: otitis media (4/5, Figure 7A, B), rhinitis (2/5, Figure 7C, D), and bronchopneumonia with bronchiectasis (3/5, Figure 7E, F). Additionally, mild bilateral hydrocephalus of the third ventricle was also seen in some females (2/5, Figure 7G, H). In FDH model mice the pneumonia was characterized by pyogranulomatous inflammation centered on foreign material (hair, food, and bedding) and colonies of coccoid bacteria within the bronchioles, consistent with aspiration pneumonia (Figure 7I, J). Aspiration pneumonia is rare in mice and its presence only in mutants argues against environmental or iatrogenic causes. Consistent with our findings, recurrent pneumonia has been reported in some FDH patients in association with gastroesophageal reflux and nasal regurgitation during feeding [27,29]. It is not known if there are lung defects that are associated with this symptom in humans. Mild right ventricular hypertrophy of the heart was observed in some mice (Figure 7K, L), which is likely secondary to pulmonary hypertension associated with pneumonia. Consistent with the observed chronic, active pulmonary inflammation, we detected significant increases in white blood cell counts, lymphocytes, monocytes and neutrophils (Figure 7Q-T). We furthermore detected an increase in red blood cell counts (Figure 7U) and significant increases in total hemoglobin concentration (Figure 7V). Whether these increases are due to dehydration or an adaptive response to poor lung function is not clear.

To investigate the cause of the bronchopneumonia, we performed Periodic Acid-Schiff (PAS) staining and histological analyses on tracheae of adult animals. We observed marked goblet cell hyperplasia as evidenced by increased numbers of PAS positive cells within the bronchioles, notably in areas of inflammation (Figure 7M, N). The PAS stain also indicated excessive mucous within lower airways. In contrast to controls (Figure 7O), the normal ciliated epithelial morphology was disrupted by segmental loss of ciliated epithelial cells (up to 200 um in length) in all adult females affected by bronchopneumonia (3/3, Figure 7P). In these segments the tracheal epithelium is replaced by single cell layered or disorganized multilayered non-ciliated cuboidal to squamous type epithelium. These changes in the tracheal epithelium of the mutants could constitute reparative and protective morphological modifications in response to chronic active inflammation. It is however possible that the segmental absence of normal ciliated epithelium might have resulted in suboptimal mucociliary function and subsequent aspiration pneumonia. It is unknown if similar defects in ciliated epithelia underlie the pneumonia observed in some human FDH patients.

Similar to the wide spectrum of human FDH symptoms, all adult *Porcn* ^*+/del*^ animals presented with an individual subset of lesions, likely due to individual XCI patterns. The spectrum of lesions observed in our FDH mouse model, namely: otitis media, rhinitis, aspiration bronchopneumonia, and hydrocephalus, have been linked to ciliopathy in humans [30] as well as some mouse models of motile cilia dysfunction [31]. While individually variable, this spectrum of lesions suggests defects in cilia function. Whether *Porcn* mutants exhibit defects in ciliogenesis or lack ciliated cells in the affected tissues due to differentiation defects remains to be tested. 

## Discussion

Wnt ligands have been shown to play numerous and redundant roles in mammalian embryonic development. As all Wnts are dependent on Porcn function [2], this protein represents a bottleneck for the entire pathway. In this study, we have used zygotic mutation of the paternal *Porcn* allele to ablate Wnt ligand secretion in embryonic development in an XCI-dependent pattern. Using this approach, we have created a mouse model for the human disease FDH. While several aspects of human FDH have been recapitulated in mice using tissue-specific *Porcn* deletions and chimera formation [14,15,17], this study presents the first zygotic heterozygous female mice that have been followed throughout embryonic development and into adulthood. 

Similar to a previous report describing an embryo-specific deletion of the maternal *Porcn* allele [15], we have encountered variable defects throughout fetal development and a dramatic perinatal lethality of 95% of zygotic paternal allele mutant heterozygous females. The variability of fetal defects can be attributed to XCI patterns that are unique and potentially skewed in each female. The individual phenotypes are thus dependent on which cells or tissues are functionally affected. This situation is further complicated by non-cell-autonomy of Wnt-related effects, as the actual phenotype may be observed in a functionally wild-type cell due to the genetic ablation of its nearby Wnt source. While the fetal lethality is not significant, a large majority of heterozygous females dies perinatally due to diaphragmatic hernias, body wall closure defects and severe kidney defects. *Porcn* heterozygous female mice surviving the perinatal period develop fairly normally, but recapitulate typical skin defects, reduced bone mineral density and also the more rare occurrence of a split sternum, which has also been observed in human patients [27].

Surprisingly, adult *Porcn* ^*+/del*^ animals display several phenotypes that are frequently associated with ciliary defects: aspiration pneumonia with bronchiectasis, rhinitis, otitis media, kidney defects and mild hydrocephalus. The accumulation of these phenotypes leads us to speculate that Porcn might be required for the differentiation of ciliated cells types or involved in the formation of functional cilia. Such an effect might be mediated by Wnt ligands, but could also be a Wnt-unrelated function. Wnt-unrelated *Porcn* functions have been reported previously [32], but remain difficult to investigate *in vivo* due to the co-occurrence of severe Wnt-related defects.

Human FDH is considered a rare syndrome, as the reported prevalence for FDH is <1/1,000,000 based on the number of observed live-births [33]. Data on human pre-natal FDH however is lacking. The data from our mouse model suggests that, similar to *Porcn* mutant male embryos, a large proportion (~95%) of heterozygous female fetuses die *in utero* or perinatally. If this extent of lethality is replicated in humans, the actual prevalence of female fetal FDH could be 20 times higher at 1/50,000 pregnancies. Moreover, when pregnancies with *PORCN* mutant male embryos are included, the overall prevalence of embryonic/fetal FDH may be up to 1/25,000 pregnancies. Supporting this estimate, the same prevalence has been observed in X-linked Charcot-Marie-Tooth disease (CMTX1, OMIM#302800, [34]), which is also an X-linked dominant disorder, but is not associated with pre-natal lethality that would mask the prevalence. Based on the mouse data presented here and by others [15,17], approximately 98% of pregnancies with embryonic *PORCN* mutations would result in lethality, as all mutant male embryos die during gastrulation and 95% of the female fetuses die during fetal development or perinatally. Consistent with these findings, fetal FDH-related perinatal lethality has also been reported in humans [35] and some studies suggest that only mildly affected patients survive beyond birth [36]. It is thus possible that FDH is actually not a rare syndrome, but has a greater impact on human pregnancies and maternal health than previously appreciated.

In summary, our analysis of adult *Porcn* ^*+/del*^ female mice has confirmed similarities between the mouse and human phenotypes, including body wall closure, skeletal, and skin defects. It has furthermore revealed a novel aspect of the phenotype, highlighted by defects in mucociliary clearance of the airways. This aspect may represent a species-specific feature or may alternatively have been missed in reported human cases. Detailed phenotypic analyses in airway-specific *Porcn* mutant mouse models and careful characterization of the human phenotypes should allow these scenarios to be distinguished. In conclusion, our mouse model provides novel insights into the etiology of FDH and may thus contribute to the improved treatment of human FDH patients.

## Materials and Methods

### Ethics Statement

All animal work was carried out following Canadian Council on Animal Care (CCAC) guidelines for Use of Animals in Research and Laboratory Animal Care under protocols approved by the Toronto Centre for Phenogenomics Animal Care Committee (ACC); animal use protocol 13-05-0026-H. 

### Mouse alleles and genetic background

All animal experiments were performed in a specific pathogen free environment at the Toronto Centre for Phenogenomics (TCP). In order to identify female mice and *Porcn* mutant cells in heterozygous animals, we established a mouse line carrying a *Porcn floxed* allele [16] in cis to the X-linked *D4/XEGFP* transgene (Tg(GFPX)4Nagy) [37] on an outbred ICR background. Both hemizygous and heterozygous animals are viable, fertile, and did not display any obvious defects. *Porcn* ^*+/del*^ female fetuses and adults were generated by crossing *Porcn* ^*lox/Y*^; *XEFP* ^*tg/Y*^ males to *pCX-NLS-Cre* ^*+/tg*^ females. In this setting, the floxed allele is deleted in all zygotes due to inheritance of the maternal Cre allele or maternal loading of the Cre transcript respectively. Female fetuses and newborns were identified by expression of the GFP transgene. Control females for adult phenotyping were generated by crossing *XEFP* ^*tg/Y*^ males to *pCX-NLS-Cre* ^*+/tg*^ females.

### Genotyping of mice and fetuses

Genotyping of mice and embryos was performed as previously described [16]. Further, fluorescent (GFP) labeling of females and PCR genotyping for Sry were used to determine the sex of fetuses.

### Staging and Imaging

Fetuses were generated by timed mating. The day of finding a vaginal plug was designated embryonic day 0.5 (E0.5) and fetuses were dissected in PBS at the indicated stages. Fetuses older than E15.5 were euthanized by decapitation and imaged on a MZ16F microscope (Leica) equipped with a MicroPublisher 5.0 RTV camera (Qimaging).

### Modified SHIRPA

The general appearance and behavior screening was performed using a modified SHIRPA protocol [38] with details at www.CMHD.ca. A 20 kHz clickbox (MRC Institute of Hearing, Nottingham, UK) was used to elicit the Preyer reflex indicative of normal hearing. Eyes were scanned for abnormalities using a pen light to reveal opacities and to assess pupillary light reflex. Extended observation and handling was used to detect gait abnormalities and/or limb weakness.

### Hematology and blood biochemistry

Blood was collected in 200 ul EDTA-coated capillary tubes prior to euthanasia. Samples were analyzed using a Hemavet Hematology Analyzer (950FS). Biochemical analysis was performed by IDEXX Reference Laboratories (Markham, ON) using a Roche Hitachi 917 Chemistry Analyzer.

### Urinalysis

Mouse urine was collected from conscious, restrained mice and analyzed using Chemstrip 4MD urinalysis test strips (Roche Diagnostics, Laval, Quebec).

### Bone mineral density analysis

Dual energy X-ray absorptiometry was performed using a PIXImus small animal densitometer (Lunar; GE Medical System, WI). Mice were anaesthetised using 5% isoflurane with 700 mL/min oxygen, and placed in prone position on the specimen tray using 2% isoflurane with 700 mL/min oxygen to maintain anaesthesia. Following whole body scanning, bone mineral content (BMC), bone area and bone mineral density (BMD) were measured, with the skull excluded from results. 

### Faxitron analysis

A high-resolution digital X-ray was taken at a magnification factor of 1.0 at 26 kVp using a Faxitron model MX-20 Specimen Radiography System with a digital camera attachment (Faxitron X-ray Corporation, IL) to determine bone structure. The images were captured on the Specimen Imaging program in the format of Digital Imaging and Communications in Medicine (DICOM) files for analysis purposes. The images were also saved as JPEG files for general viewing purposes.

### Necropsy and histology

Adult female mice were euthanized at 9-10 weeks of age by CO_2_. A standard panel of organs and tissues were collected and fixed by immersion (1:10 volume) in 10% neutral buffered formalin for 48 hours before transfer to 70% Ethanol. Tissues were embedded in paraffin and sectioned at 4μm for routine Hematoxylin and Eosin (H&E) staining. Lung tissues were additionally stained with Periodic acid-Schiff (PAS) stain. E18.5 embryos were removed by cesarean section, euthanized by decapitation, and fixed by immersion in buffered formalin for 48 hours. Fetuses were embedded in paraffin, midsagittal and parasagittal sections were made and routinely stained with H&E.

### Immunohistochemistry

Tissue sections were deparaffinized, rehydrated, and antigens were retrieved by Pepsin treatment at room temperature for 10 minutes. Endogenous peroxidase activity was quenched by 3% hydrogen peroxide treatment. After blocking, GFP was detected using anti-GFP rabbit IgG (Invitrogen, A11122), Elite ABC Kit (Vectastain, PK-6101) and DAB Peroxidase Substrate Kit (Vectastain, SK-4100) according to manufacturer’s instructions.
